# Solving the master equation for Indels

**DOI:** 10.1186/s12859-017-1665-1

**Published:** 2017-05-12

**Authors:** Ian H. Holmes

**Affiliations:** Dept of Bioengineering, University of California, Berkeley, 94720 USA

**Keywords:** Phylogenetics, Alignment, Indels

## Abstract

**Background:**

Despite the long-anticipated possibility of putting sequence alignment on the same footing as statistical phylogenetics, theorists have struggled to develop time-dependent evolutionary models for indels that are as tractable as the analogous models for substitution events.

**Main text:**

This paper discusses progress in the area of insertion-deletion models, in view of recent work by Ezawa (BMC Bioinformatics 17:304, 2016); (BMC Bioinformatics 17:397, 2016); (BMC Bioinformatics 17:457, 2016) on the calculation of time-dependent gap length distributions in pairwise alignments, and current approaches for extending these approaches from ancestor-descendant pairs to phylogenetic trees.

**Conclusions:**

While approximations that use finite-state machines (Pair HMMs and transducers) currently represent the most practical approach to problems such as sequence alignment and phylogeny, more rigorous approaches that work directly with the matrix exponential of the underlying continuous-time Markov chain also show promise, especially in view of recent advances.

## Background

Models of sequence evolution, formulated as continuous-time discrete-state Markov chains, are central to statistical phylogenetics and bioinformatics. As descriptions of the process of nucleotide or amino acid substitution, their earliest uses were to estimate evolutionary distances [1], parameterize sequence alignment algorithms [2], and construct phylogenetic trees [3]. Variations on these models, including extra latent variables, have been used to estimate spatial variation in evolutionary rates [4, 5]; these patterns of spatial variation have been used to predict exon structures of protein-coding genes [6, 7], foldback structure of non-coding RNA genes [8, 9], regulatory elements [10], ultra-conserved elements [11], protein secondary structures [12], and transmembrane structures [13]. They are widely used to reconstruct ancestral sequences [14–22], a method that is finding increasing application in synthetic biology [16, 20–22]. Trees built using substitution models used to classify species [23], predict protein function [24], inform conservation efforts [25], or identify novel pathogens [26]. In the analysis of rapidly evolving pathogens, these methods are used to uncover population histories [27], analyze transmission dynamics [28], reconstruct key transmission events [29], and predict future evolutionary trends [30]. There are many other applications; the ones listed above were selected to give some idea of how influential these models have been.

Continuous-time Markov chains describe evolution in a state space *Φ*, for example the set of nucleotides *Φ*={A,C,G,T}. The stochastic process *ϕ*(*t*) at any given instant of time, *t*, takes one of the values in *Φ*. Let $\vec {p}(t)$ be a vector describing the marginal probability distribution of the process at a single point in time: $\vec {p}_{i}(t) = P(\phi (t) = i)$. The time-evolution of this vector is governed by a *master equation*

1 $$ \frac{d}{dt} \vec{p}(t) = \vec{p}(t) \mathbf{R}  $$

where, for *i,j*∈*Φ* and *i*≠*j*, **R**_*i,j*_ is the instantaneous rate of mutation from state *i* to state *j*. For probabilistic normalization of Eq. 1, it is then required that

$$\mathbf{R}_{i,i} = -\sum_{j \in \Phi, j \neq i} \mathbf{R}_{i,j} $$

The probability distribution of this process at equilibrium is given by the vector $\vec {\pi }$, which must satisfy the equation

$$\vec{\pi} \mathbf{R} = \vec{0} $$

The general solution to Eq. 1 can be written $\vec {p}(t) = \vec {p}(0) \mathbf {M}(t)$ where **M**(*t*) is the *matrix exponential*

2 $$ \mathbf{M}(t) = \exp (\mathbf{R} t)   $$

Entry **M**_*i,j*_(*t*) of this matrix is the probability *P*(*ϕ*(*t*)=*j*|*ϕ*(0)=*i*) that, conditional on starting in state *i*, the system will after time *t* be in state *j*. It follows, by definition, that this matrix satisfies the Chapman-Kolmogorov forward equation:

3 $$ \mathbf{M}(t) \mathbf{M}(u) = \mathbf{M}(t+u)  $$

That is, if **M**_*i,j*_(*t*) is the probability that state *i* will, after a finite time interval *t*, have evolved into state *j*, and **M**_*j,k*_(*u*) is the analogous probability that state *j* will after time *u* evolve into state *k*, then summing out *j* has the expected result:

$$\sum_{j \in \Phi} \mathbf{M}_{i,j}(t) \mathbf{M}_{j,k}(u) = \mathbf{M}_{i,k}(t+u) \quad \forall i,k \in \Phi $$

This is one way of stating the defining property of a Markov chain: its lack of historical memory previous to its current state. Equation 1 is just an instantaneous version of this equation, and Eq. 3 is the same equation in matrix form.

The conditional likelihood for an ancestor-descendant pair can be converted into a phylogenetic likelihood for a set of extant taxon states *S* related by a tree *T*, as follows. (I assume for convenience that *T* is a binary tree, though relaxing this constraint is straightforward.)

To compute the likelihood requires that one first computes, for every node *n* in the tree, the probability $\vec {F}_{i}(n)$ of all observed states at leaf nodes descended from node *n*, conditioned on node *n* being in state *i*. This is given by Felsenstein’s *pruning recursion*:

4 $$ {\vec{F}(n) \,=\,\! \left\{\!\! \begin{array}{ll} \left(\!\mathbf{M}(t_{nl}) \vec{F}(l)\! \right)\! \circ\! \left(\! \mathbf{M}(t_{nr}) \vec{F}(r)\! \right) & \text{if } {n} \text{ is an internal node with children } l,r \\ \vec{\Delta}(s_{n}) & \text{if } {n} \text{ is a leaf node in state } s_{n} \end{array} \right.}  $$

where *t*_*mn*_ denotes the length of the branch from tree node *m* to tree node *n*. I have used the notation $\vec {\Delta }(j)$ for the unit vector in dimension *j*, and the symbol ∘ to denote the *Hadamard product* (also known as the *pointwise product*), defined such that for any two vectors $\vec {u},\vec {v}$ of the same size: $(\vec {u} \circ \vec {v})_{i} = \vec {u}_{i} \vec {v}_{i}$.

Supposing that node 1 is the root node of the tree, and that the distribution of states at this root node is given by $\vec {\rho }$, the likelihood can be written as

5 $$ P(S|T,\mathbf{R},\vec{\rho}) = \vec{\rho} \cdot \vec{F}(1)  $$

where $\vec {u} \cdot \vec {v}$ denotes the scalar product of $\vec {u}$ and $\vec {v}$. It is common to assume that the root node is at equilibrium, so that $\vec {\rho } = \vec {\pi }$.

As mentioned above, this mathematical approach is fundamental to statistical phylogenetics and many applications in bioinformatics. For small state spaces *Φ*, such as (for example) the 20 amino acids or 61 sense codons, the matrix exponential **M**(*t*) in Eq. 2 can be solved exactly and practically by the technique of spectral decomposition (i.e. finding eigenvalues and eigenvectors). Such an approach informs the Dayhoff PAM matrix. It was also solved for certain specific parametric forms of the rate matrix **R** by Jukes and Cantor [1], Kimura [31], Felsenstein [3], and Hasegawa et al. [32], among others. This approach is used by all likelihood-based phylogenetics tools, such as RevBayes [33], BEAST [34], RAxML [35], HyPhy [36], PAML [37], PHYLIP [38], TREE-PUZZLE [39], and XRate [40]. Many more bioinformatics tools use the Dayhoff PAM matrix or other substitution matrix based on an underlying master equation of the form Eq. 1.

### Homogeneity, stationarity, and reversibility

There exists a deep literature on Markov chains, to which this brief survey cannot remotely do justice, but several concepts must be mentioned in order to survey progress in this area.

A Markov chain is *time-homogeneous* if the elements of the rate matrix **R** in Eq. 1 are themselves independent of time. If a Markov chain is time-homogeneous and is known to be in equilibrium at a given time, for example $\vec {p}(0) = \vec {\pi }$, then (absent any other constraints) it will be in equilibrium at all times; such a chain is referred to as being *stationary*.

The time-scaling of these models is somewhat arbitrary: if the time parameter *t* is replaced by a scaled version *t*/*κ*, while the rate matrix **R** is replaced by **R** *κ*, then the likelihood in Eq. 2 is unchanged. For some models, the rate is allowed to vary between sites [4, 5].

A Markov chain is *reversible* if it satisfies the instantaneous *detailed balance* condition $\vec {\pi }_{i} \mathbf {R}_{i,j} = \vec {\pi }_{j} \mathbf {R}_{j,i}$, or its finite-time equivalent $\vec {\pi }_{i} \mathbf {M}_{i,j} = \vec {\pi }_{j} \mathbf {M}_{j,i}$. This amounts to a symmetry constraint on the parameter space of the chain (specifically, the matrix **S** with elements $\mathbf {S}_{i,j} = \sqrt {\vec {\pi }_{i} / \vec {\pi }_{j}} \mathbf {R}_{i,j}$ is symmetric) which has several convenient advantages: it effectively halves the number of parameters that must be determined, it eases some of the matrix manipulations (symmetric matrices have real eigenvalues and the algorithms to find them are generally more stable), and it allows for some convenient manipulations, such as the so-called *pulley principle* allowing for arbitrary re-rooting of the tree [3]. From another angle, however, these supposed advantages may be viewed as drawbacks: reversibility is a simplification which ignores some unreversible aspects of real data, limits the expressiveness of the model, and makes the root node placement statistically unidentifiable.

Stationarity has similar advantages and drawbacks. If one assumes the process was started at equilibrium, that is one less set of parameters to worry about (since the equilibrium distribution is implied by the process itself), but it also renders the model less expressive and makes some kinds of inference impossible.

The early literature on substitution models involved generalizing from rate matrices **R** characterized only by a single rate parameter [1], to symmetry-breaking versions that allowed for different transition and transversion rates [31], non-uniform equilibrium distributions over nucleotides [3], and combinations of the above [32]. These models are all, however, reversible. A good deal of subsequent research has gone into the problem, in various guises, of generalizing results obtained for reversible, homogeneous and/or stationary models to the analogous irreversible, nonhomogeneous and nonstationary models. For examples, see [30, 41–43].

### From individual residues to whole sequences

The question naturally arises: how to extend the model to describe the evolution of an entire sequence, not just individual sites? In such cases, when one talks about **M**(*t*)_*i,j*_ “the likelihood of an ancestor-descendant pair (*i,j*)” (or, more precisely, the probability that—given the system starts in ancestral state *i*—it will after time *t* have evolved into descendant state *j*) one must bear in mind that the states *i* and *j* now represent not just individual residues, but entire sequences.

As long as the allowed mutations are restricted to point substitutions and their mutation rates are independent of flanking context, then the extension to whole sequences is trivially easy: one can simply multiply together probabilities of independent sites.

However, many kinds of mutation violate this assumption of site independence; most notably context-dependent substitutions and indels, where the rates depend on neighboring sites. For these mutations the natural approach is to extend the state space *Φ* to be the set of all possible sequences over a given alphabet (for example, the set of all DNA or protein sequences). This state space is (countably) infinite; Eqs. 1-4 can still be used on an infinite state space, but solution by brute-force enumeration of eigenvalues and eigenvectors is no longer feasible, except in special cases where there is explicit structure to the rate matrix that allows identification of the eigensystem by algebraic approaches [44, 45].

It has turned out that whole-sequence evolutionary models have proved quite challenging for theorists. There is extensive evidence suggesting that indels, in particular, can be profoundly informative to phylogenetic studies, and to applications of phylogenetics in sequence analysis [46–51]. The field of efforts to unify alignment and phylogeny, and to build a theoretical framework for the evolutionary analysis of indels, has been dubbed *statistical alignment* by Hein, one of its pioneers [52]. Recent publications by Ezawa [53–55] and Rivas and Eddy [56] have highlighted this problem once again, directly leading to the present review.

## Main text

In this paper I focus only on “local” mutations: mostly indel events (which may include local duplications), but also context-dependent substitutions. This is not because “nonlocal” events (such as rearrangements) are unimportant, but rather that they tend to defy phylogenetic reconstruction due to the rapid proliferation of possible histories after even a few such events [57].

The discussion here is separated into two parts. In the first part, I discuss the master equation (Eq. 1) and exact solutions thereof (Eq. 2), along with various approximations and their departure from the Chapman-Kolmogorov ideal (Eq. 3). This is an area in which recent progress has been reported in this journal. In the second part, I review the extension from pairwise probability distributions to phylogenetic likelihoods of multiple sequences, using analogs of Felsenstein’s pruning recursion (Eq. 4).

### Solving the master equation

This section begins with various approaches to finding the time-dependent probability distribution of gap lengths in a pairwise alignment, under several evolutionary models.

#### Exactly solved models on k-mer strings

As an approach to models on strings of unbounded length, one can consider short motifs of *k* residues. These can still be considered as finite state-space models; for example, a *k*-nucleotide model has 4^*k*^ possible states. Several such models have been analyzed, including models on codons where *k*=3 [47, 58], dinucleotides involved in RNA base-pairs where *k*=2 [59–61], and models over sequences of arbitrary length *k* [44, 62].

Mostly, these models handle short sequences (motifs) and do not allow the sequence length to change over time (so they model only substitutions and not indels). Some of the later models do allow the sequence length to change via insertions or deletions [62] though these models have not yet been analyzed in a way that would allow the computation of alignment likelihoods for sequences of realistic lengths.

#### Exactly solved models on strings of unbounded length

It is a remarkable reflection on the extremely challenging nature of this problem that, to date, the only exactly solved indel model on strings is the TKF91 model, named after the authors’ initials and date of publication of this seminal paper [63]. While there has been progress in developing approximate models in the 25 years since the publication of this paper, and in extending it from pairwise to multiple sequence alignment, it remains the only model for which

1. the state space *Φ* is the set of all sequences (strings) over a finite alphabet,
2. the state space is ergodically explored by substitutions and indels (so there is a valid alignment and evolutionary trajectory between any two sequences *ϕ*(0) and *ϕ*(*t*)),
3. Equation 2 can be calculated exactly (specifically, as a sum over alignments, where the individual alignment likelihoods can be written in closed form).

The TKF91 model allows single-residue context-independent events only. These include (i) single-residue substitutions, (ii) single-residue insertions (with the inserted residue drawn from the equilibrium distribution of the substitution process), and (iii) single-residue deletions (whose rates are independent of the residue being deleted). The rates of all these mutation events are independent of the flanking sequence.

This process is equivalent to a linear birth-death model with constant immigration [64]. Thorne et al. showed that an ancestral sequence can be split into independently evolving zones, one for each ancestral residue (or “links”, as they call them). This leads to the very appealing result that the length distribution for observed gaps is geometric, which conveniently allows the joint probability *P*(*ϕ*(0),*ϕ*(*t*)) to be expressed as a paired-sequence Hidden Markov Model or “Pair HMM” [65]. The conditional probability *P*(*ϕ*(*t*)|*ϕ*(0)) can similarly be expressed as a weighted finite-state transducer [66–68]. Some interesting discussion of why the TKF91 model should be solvable at all can be found in [69] and in [42].

There are several variations on the TKF91 model. The case where there are no indels at all, only substitutions, can be viewed as a special case of TKF91, and can of course be solved exactly, as is well known. Another variation on the TKF91 model constrains the total indel rate to be independent of sequence length [70].

In the following section I cover some variants that use different state spaces.

#### Exactly solved models on state spaces other than strings

It is difficult to extend TKF91 to more realistic models wherein indels (or substitutions) can affect multiple residues at once. In such models, the fate of adjacent residues is no longer independent, since a single event can span multiple sites.

As a way around this difficulty, several researchers have developed evolutionary models where the state is not a pure DNA or protein sequence, but includes some extra “hidden” information, such as boundaries, markers or other latent structure. In some of these models the sequence of residues is replaced by a sequence of indivisible fragments, each of which can contain more than one residue [56, 69, 71]. These includes the TKF92 model [71] which is, essentially, TKF91 with residues replaced by fragments (so the alphabet itself is the countably infinite set of all sequences over some other, finite alphabet). Other models approximate indels as a kind of substitution that temporarily hides a residue, by augmenting the DNA or protein alphabet with an additional gap character [72–74].

These models can be used to calculate some form of likelihood for a pairwise alignment of two sequences, but since this likelihood is not derived from an underlying instantaneous model of indels, the equations do not, in general, satisfy the Chapman-Kolmogorov forward Eq. (3). That is, the probability of evolving from *i* to *k* comes out differently depending on whether or not one conditions on an intermediate sequence *j*. Clearly, something about this “seems wrong”: the failure to obey Eq. 3 illustrates the ad hoc nature of these approaches. Ezawa [53] describes the Chapman-Kolmogorov property (Eq. 3) as *evolutionary consistency*; it can also be regarded as being the defining property of any correct solution to a continuous-time Markov chain. The above-mentioned approaches may be evolutionarily consistent if the state space is allowed to include the extra information that is introduced to make the model tractable, such as fragment boundaries. Lèbre and Michel have criticized other aspects of the Rivas-Eddy 2005 and 2008 models [73, 74]; in particular, incomplete separation of the indel and substitution processes [42].

Models which allow for heterogeneity of indel and substitution rates along the sequence also fall into this category of latent variable models. The usual way of allowing for such spatial variation in substitution models is to assume a latent rate-scaling parameter associated with each site [4, 5]. For indel models, this latent information must be extended to include hidden site boundaries [56].

#### Exactly solved models on graphs

Another variation on TKF91 is the TKF Structure Tree, which describes the evolutionary behavior of RNA structures with stem and loop regions which are subject to insertion and deletion [75]. Rather than describing the evolution of a sequence, this model essentially captures the time-evolution grammar of a tree-like graph whose individual edges are evolving according to the TKF91 model. Other evolutionary models have made use of graph grammars, for example to model pseudoknots [76] or context-dependent indels [77].

#### Finite-event trajectories

In tackling indel models where the indel events can insert or delete multiple residues at once, several authors have used the approximation that indels never overlap, so that any observed gap corresponds to a single indel event. This approximation, which is justified if one is considering evolutionary timespans *t*≪1/(*δ* *ℓ*) where *δ* is the indel rate per site and *ℓ* is the gap length, considerably simplifies the task of calculating gap probabilities [67, 78–83].

At longer timescales, it is necessary to consider multiple-event trajectories, but (as a simplifying approximation) one can still truncate the trajectory at a finite number of events. A problem with this approach is that many different trajectories will generally be consistent with an observed mutation. Summing over all such trajectories, to compute the probability of observing a particular configuration after finite time (e.g. the observed gap length distribution), is a nontrivial problem.

In analyzing the *long indel* model, a generalization of TKF91 with arbitrary length distributions for instantaneous indel events, Miklós et al. [84] make the claim that the existence of a conserved residue implies the alignment probability is factorable at that point (since no indel has ever crossed the boundary). They use a numerical sum over indel trajectories to approximate the probability distribution of observed gap lengths. Although they used a reversible model, their approach generalizes readily to irreversible models. This work builds on an earlier model which allows long insertions, but only single-residue deletions [85]. Recent work by Ezawa has put this finite-event approximation on a more solid footing by developing a rigorous algebraic definition of equivalence classes for event trajectories [53–55].

Solutions obtained using finite-event approximations will not exactly satisfy Eq. 3. There will be some error in the probability, and in general the error will be greater on longer branches, as the main assumption behind the approximation (that there are no overlapping indels in the time interval, or that there is a finite limit to the number of overlapping indels) starts to break down. However, since these are principled approximations, it should be possible to form some conclusions as to the severity of the error, and its dependence on model parameters. Simulation studies have also been of some help in assessing the error of these approximations.

#### Taylor series approximations

For context-dependent substitution processes, such as models that include methylation-induced CpG-deamination, a clever approach was developed in [44]. Rather than considering a finite-event trajectory, they develop an explicit Taylor series for the matrix exponential (Eq. 2) and then truncate this Taylor series. Specifically, the rate matrix for a finite-length sequence is constructed as a sum of rate matrices operating locally on the sequence, using the Kronecker sum ⊕ and Kronecker product ⊗ to concatenate rate matrices. These operators may be understood as follows, for an alphabet of *N* symbols: suppose that ${\mathcal {M}}_{m}$ is the set of all matrices indexed by *m*-mers, so that if $\mathbf {A} \in {\mathcal {M}}_{m}$, then **A** is an *N* ^*m*^×*N* ^*m*^ matrix. Let *i,j* be *m*-mers, *k,l* be *n*-mers, and *ik, jl* the concatenated *m*+*n*-mers. If $\mathbf {A} \in {\mathcal {M}}_{m}$ and $\mathbf {B} \in {\mathcal {M}}_{n}$ then **A**⊕**B** and **A**⊗**B** are both in ${\mathcal {M}}_{m+n}$ and are specified by

6 $$\begin{array}{@{}rcl@{}} \left(\mathbf{A} \otimes \mathbf{B} \right)_{ik,jl} & = & \mathbf{A}_{i,j} \mathbf{B}_{k,l} \end{array} $$

7 $$\begin{array}{@{}rcl@{}} \left(\mathbf{A} \oplus \mathbf{B} \right)_{ik,jl} & = & \delta(k=l) \mathbf{A}_{i,j} + \delta(i=j) \mathbf{B}_{k,l} \end{array} $$

where *δ*(*i*=*j*)=1 if *i*=*j* and 0 if *i*≠*j*. Furthermore, suppose $\mathbf {O}_{n} \in {\mathcal {M}}_{n}$ is the *N* ^*k*^×*N* ^*k*^ null matrix containing only zeroes. Then **O**_*m*_⊕**B** commutes with **A**⊕**O**_*n*_, and exp(**A**⊕**B**)= exp(**A**)⊗ exp(**B**). The rate matrix **R** for a length-*L* sequence operated on locally by a context-sensitive rate matrix $\mathbf {A} \in {\mathcal {M}}_{m}$ can be written as a sum of the form

$$\mathbf{R} = \sum_{n=0}^{L-m} \mathbf{O}_{n} \oplus \mathbf{A} \oplus \mathbf{O}_{L-m-n} $$

Commuting terms in the Taylor series for exp(**R** *t*) can then be systematically rearranged into a quickly-converging dynamic programming recursion. This approach was first used by [44] and further developed including model-fitting algorithms [86] application to phylogenetic trees [87] and discussion of the associated eigensystem [45, 62]. It remains to be seen to what extent such an approach offers a practical solution for general indel models, where the instantaneous transitions are between sequences of differing lengths.

#### Simulation studies

Such is the difficulty of solving long indel models that several authors have performed simulations to investigate the empirical gap length distributions that are observed after finite time intervals for various given instantaneous indel-rate models. These observed gaps can arise from multiple overlapping indel events, in ways that have so far defied straightforward algebraic characterization.

In recent work, Rivas and Eddy [56] have shown that if an underlying model has a simple geometric length distribution over instantaneous indel events, the observed gap length distribution at finite times (accounting for the possibility of multiple, overlapping indels) cannot be geometric. Rivas and Eddy report simulation studies supporting this result, and go on to propose several models incorporating hidden information (such as fragment boundaries, *a la* TKF92) which have the advantage of being good fits to HMMs for their finite-time distributions.

It has long been known that the lengths of empirically-observed indels are more accurately described by a power-law distribution than a geometric distribution [46, 47, 88–91] and that alignment algorithms may benefit from using such length distributions, which lead to generalized convex gap penalties, rather than the computationally more convenient geometric distribution, which leads to an affine or linear gap penalty [92, 93]. For molecular evolution purposes in particular, it is known that overreliance on affine gap penalties leads to serious underestimates of the true lengths of natural indels [94]. For almost as long, it has been known that using a mixture of geometric distributions, or (considered in score space rather than probability space) a piecewise linear gap penalty, mitigates some of these problems in sequence alignment [94–96]. Taken together, these results suggest that simple HMM-like models, which are most efficient at modeling geometric length distributions, may be fundamentally limited in their ability to fully describe indels; that adding more states (yielding length distributions that arise from sums of geometric random variates, such as negative binomial distributions, or mixtures of geometric distributions) can lead to an improvement; and that generalized HMMs, which can model arbitrary length distributions at the cost of some computational efficiency [97], may be most appropriate. For example, the abovementioned “long indel” model of Miklós et al. uses a generalized Pair HMM [84], as does the HMM of [98]. It is even conceivable that some molecular evolution studies in the future will abandon HMMs altogether, although they remain very convenient for many applications.

The recent work of Ezawa has some parallels, but also differences, to the work of Rivas and Eddy [53–55]. Ezawa criticizes over-reliance on HMM-like models, and insists on a systematic derivation from simple instantaneous models. He puts the intuition of Miklós et al. [84] on a more formal footing by introducing an explicit notation for indel trajectories and the concept of “local history set equivalence classes” for equivalent trajectories. Ezawa uses this concept to prove that alignment likelihoods for long-indel and related models are indeed factorable, and investigates, by numerical computation and analysis (with confirmation by simulation), the relative contribution of multiple-event trajectories to gap length distributions. Ezawa’s results also show that the effects on the observed indel lengths due to overlapping indels become more significant as the indels get larger, making the problem particularly acute for genomic alignments where indels can be much larger than in proteins.

A number of excellent, realistic sequence simulators are available including DAWG [99], INDELible [100], and indel-Seq-Gen [101].

### Extending from pairs to trees

Consider now the extension of these results from pairwise alignments, such as TKF91 and the “long indel” model, to multiple alignments (with associated phylogenies). Some of the approaches to this problem use Markov Chain Monte Carlo (MCMC); some of the approaches use finite-state automata; and there is also some overlap between these categories (i.e. MCMC approaches that use automata).

#### Approaches based on MCMC sampling

MCMC is the most principled approach to integrating phylogeny with multiple alignment. In principle an MCMC algorithm for phylogenetic alignment can yield the posterior distribution of alignments, trees, and parameters for any model whose pairwise distribution can be computed. This includes long indel models and also, in principle, other effects such as context-dependent substitutions.

Of the MCMC methods reported in the literature, some just focus on alignment and ancestral sequence reconstruction [65]; others on simultaneous alignment and phylogenetic reconstruction [79–81, 83, 102, 103]; some also include estimation of evolutionary parameters such as *dN*/*dS* [104]; and some (focused on RNA sequences) attempt prediction of secondary structure [105, 106].

In practise these mostly use HMMs, or dynamic programming of some form, in common with the methods of the following section. It is of course possible to use HMM-based or other MCMC approaches to propose candidate reconstructions, and then to accept or reject those proposals (in the manner of Metropolis-Hastings or importance sampling) using a more realistic formulation of the indel likelihood. Ezawa’s methods, and others that build on them or are related to them, may be useful in this context. For example, Ezawa’s formulation was used to calculate the indel component of the probability of a fixed multiple sequence alignment (MSA) resulting from sequence evolution along a fixed tree [53]. He also developed an algorithm to approximately calculate the indel component of the MSA probability using all MSA-compatible parsimonious indel histories [54], and applied it to some analyses of simulated MSAs [107]. Using such realistic likelihood calculations as a post-processing “filter” for coarser, more rapid MCMC approaches that sample the space of possible reconstructions could be a promising approach.

#### Approaches based on automata theory

The dynamic programming recursion for pairwise alignment reported for the TKF91 model [63] can be exactly extended to alignment of multiple sequences given a tree [108, 109]. This works essentially because the TKF91 joint distribution over ancestor and descendant sequences can be represented as a Pair HMM; the multiple-sequence version is a multi-sequence HMM [65].

This approach can be generalized, using *finite-state transducer* theory. Transducers were originally developed as modular representations of sequence-transforming operations for use in speech recognition [110]. In bioinformatics, they offer (among other things) a systematic way of extending HMM-like pairwise alignment likelihoods to trees [67, 68, 111, 112]. Other applications of transducer models in bioinformatics have included copy number variation in tumors [113], protein family classification [114], DNA-protein alignment [115] and error-correcting codes for DNA storage [116].

A finite-state transducer is a state machine that reads an input tape and writes to an output tape [117]. A probabilistically weighted finite-state transducer is the same, but its behavior is stochastic [110]. For the purposes of bioinformatics sequence analysis, a transducer can be thought of as being just like a Pair HMM; except where a Pair HMM’s transition and emission probabilities have been normalized so as to describe joint probabilities, a transducer’s probabilities are normalized so as to describe conditional probabilities like the entries of matrix **M**(*t*) (Eq. 2). More specifically, if *i* and *j* are sequences, then one can define the matrix entry *A*_*i,j*_ to be the Forward score for those two sequences using transducer ${\mathcal {A}}$. Thus, the transducer is a compact encoding for a square matrix of countably infinite rank, indexed by sequence states (rather than nucleotide or amino acid states).

The utility of transducers arises since for many purposes they can be manipulated analogously to matrices, while being more compact than the corresponding matrix (as noted above, matrices describing evolution of arbitrary-length sequences are impractically—or even infinitely—large). If ${\mathcal {A}}$ and ${\mathcal {B}}$ are finite transducers encoding (potentially infinite) matrices **A** and **B**, then there is a well-defined operation called *transducer composition* yielding a finite transducer ${\mathcal {A}}{\mathcal {B}}$ that represents the matrix product **A** **B**. There are other well-defined transducer operations corresponding to the various other linear algebra operations used in this paper: the Hadamard product (∘) corresponds to *transducer intersection*, the Kronecker product (⊗) corresponds to *transducer concatenation*, and the scalar product (·) and the unit vector ($\vec {\Delta }$) can also readily be constructed using transducers. Consequently, Eq. 4 can be interpreted directly in terms of transducers [67, 68, 82].

This has several benefits. One is theoretical unification: Eq. 4, using the above linear algebra interpretation of transducer manipulations, turns out to be very similar to Sankoff’s algorithm for phylogenetic multiple alignment [118]. Thus is a famous algorithm in bioinformatics unified with a famous algorithm in likelihood phylogenetics by using a tool from computational linguistics. (This excludes the RNA structure-prediction component of Sankoff’s algorithm; that can, however, be included by extending the transducer framework to pushdown automata [119].) Practically, the phylogenetic transducer can be used for alignment [79, 81], parameter estimation [104], and ancestral reconstruction [67], with promising results for improved accuracy in multiple sequence alignment [112].

More broadly, one can think of the transducer as being in a family of methods that combine phylogenetic trees (modeling the temporal structure of evolution) with automata theory, grammars, and dynamic programming on sequences (modeling the spatial structure of evolution). The TKF Structure Tree, mentioned above, is in this family too: it can be viewed as a context-free grammar, or as a transducer with a pushdown stack [75].

The HMM-like nature of TKF91, and the ubiquity of HMMs and dynamic programming in sequence analysis, has motivated numerous approaches to indel analysis based on Pair HMMs [56, 71, 74, 78, 120], as well as many other applications of phylogenetic HMMs [6, 7, 12, 121, 122] and phylogenetic grammars [8, 10, 40, 60, 123, 124]. In most of these models, an alignment is assumed fixed and the HMM or grammar used to partition it; however, in principle, one can combine the ability of HMMs/grammars to model indels (and thus impute alignments) with the ability to partition sequences into differently evolving regions.

## Conclusions

The promise of using continuous-time Markov chains to model indels has been partially realized by automata-theoretic approaches based on transducers and HMMs. Recent work by Rivas and Eddy [56] and by Ezawa [53–55] may be interpreted as both good and bad news for automata-theoretic approaches.

It appears that closed-form solutions for observed gap length distributions at finite times, and in particular the geometric distributions that simple automata are good at modeling, are still out of reach for realistic indel models, and indeed (for simple models) have been proven impossible [56]. Further, simulation results have demonstrated that geometric distributions are not a good fit to the observed gap length distributions when the underlying indel model has geometrically-distributed lengths for its instantaneous indel events [56]. If the lengths of the instantaneous indels follow biologically plausible power-law distributions, the evolutionary effects due to overlapping indels become larger as the gaps grow longer [54].

That is the bad news (at least for automata). The good news is that the simulation results also suggest that, for short branches and/or gaps (such that indels rarely overlap), the error may not be too bad to live with. Approximate-fit approaches that are common in Pair HMM modeling and pairwise sequence alignment—such as using a mixture of geometric distributions to approximate a gap length distribution (yielding a longer tail than can be modeled using a pure geometric distribution)—may help bridge the accuracy gap [96]. Given the power of automata-theoretic approaches, the best way forward (in the absence of a closed-form solution) may be to embrace such approximations and live with the ensuing error.

Interestingly, the authors of the two recent simulation studies that prompted this commentary come to different conclusions about the viability of automata-based dynamic programming approaches. Ezawa [53, 54], arguing that realism is paramount, advocates deeper study of the gap length distributions obtained from simple instantaneous models—while acknowledging that such gap length distributions may be more difficult to use in practice than the simple geometric distributions offered by HMM-like models. Rivas and Eddy [56], clearly targeting applications (particularly those such as profile HMMs), work backward from HMM-like models toward evolutionary models with embedded hidden information. These models may be somewhat mathematically contrived, but are easier to tailor so as to model effects such as position-specific conservation, thus trading (in a certain sense) purism for expressiveness.

Whichever approach is used, these results are unambiguously good news for the theoretical study of indel processes. The potential benefits of modeling alignment as an aspect of statistical phylogenetics are significant. One can reasonably hope that the advance of theoretical work in this area will continue to inform advances in both bioinformatics and statistical phylogenetics. After all, and in spite of the Cambrian explosion in bioinformatics sub-disciplines, sequence alignment and phylogeny truly are closely related aspects of mathematical biology.
